# Cumulative risk exposure and emotional symptoms among early adolescent girls

**DOI:** 10.1186/s12905-021-01527-7

**Published:** 2021-11-05

**Authors:** Ola Demkowicz, Margarita Panayiotou, Neil Humphrey

**Affiliations:** Manchester Institute of Education, The University of Manchester, Manchester, UK

**Keywords:** Women’s health, Adolescent mental health, Emotional symptoms, Inequality, Risk exposure, Cumulative risk

## Abstract

**Background:**

From early adolescence, girls and women report the highest rates of emotional symptoms, and there is evidence of increased prevalence in recent years. We investigate risk factors and cumulative risk exposure (CRE) in relation to emotional symptoms among early adolescent girls.

**Methods:**

We used secondary data analysis, drawing on data capturing demographic information and self-reported emotional symptoms from 8327 girls aged 11–12 years from the 2017 baseline data collection phase of the HeadStart evaluation. We used structural equation modelling to identify risk factors in relation to self-reported emotional symptoms, and collated this into a CRE index to investigate associations between CRE and emotional symptoms.

**Results:**

Four risk factors were found to have a statistically significant relationship with emotional symptoms among early adolescent girls: low academic attainment, special educational needs, low family income, and caregiving responsibilities. CRE was positively associated with emotional symptoms, with a small effect size.

**Conclusions:**

Results identify risk factors (outlined above) that are associated with emotional symptoms among early adolescent girls, and highlight that early adolescent girls experiencing a greater number of risk factors in their lives are likely to also experience greater emotional distress. Findings highlight the need for identification and targeted mental health intervention (e.g., individual or group counselling, approaches targeting specific symptoms), for those facing greater risk and/or with emergent symptoms.

## Background

In early adolescence, evidence suggests that girls begin to experience greater levels of emotional symptoms (i.e., depressive and anxious symptoms) than boys, typically around the age of 12 years[1].^1^ Studies show this disparity exists throughout the lifespan; girls and women are twice as likely to report depressive symptoms and disorder from mid-adolescence compared to boys and men [1]. They are also more likely to experience anxious symptoms and disorders, though this fluctuates based on type of anxiety [2]. Depressive and anxious symptoms are distinct but strongly inter-related, with high comorbidity rates among adolescents [3]. Research indicates a significant increase in emotional symptoms and disorder among adolescent girls in recent years, in the United Kingdom [4–7] and other Western and non-Western countries [8, 9], necessitating urgent research into the factors associated with such difficulties. These studies consistently point to apparent increases in emotional difficulties as a whole (i.e., rather than just depressive *or* anxious symptomatology) and to increases only among girls, and not among boys in the same cohorts [4–9]. Effects have been observed across different points in adolescence, starting in early adolescence [6]. Typically these increases among girls are small, but as noted by Fink et al. [6] the effect is not negligible and warrants attention.

We set out to investigate the risk factors associated with emotional symptoms among girls aged 11–12 years, given evidence that such symptoms are increasing among girls. Furthermore, as risk factors tend to co-occur [10], we also examined whether exposure to a greater number of risk factors corresponds to increased symptoms. We focused on investigating possible factors associated with symptoms among a 2017 sample of adolescent girls, offering valuable insight into epidemiological patterns and levels of exposure for a vulnerable group at a recent timepoint, rather than factors that may be contributing to an increase in such symptoms, which currently are not well understood. We focused on symptoms rather than disorder given the reported increase in general symptomatology among girls [4–7]. Furthermore, evidence indicates that depression and anxiety symptoms go beyond those specified within constricted diagnostic criteria, suggesting that psychopathology is continuous and not narrowly expressed through distinct disorders [11, 12].

Existing evidence relating to risk factors for emotional symptoms in childhood and adolescence (e.g., low family income [13]) suffers from key gaps and limitations. First, it is critical that in this area of work, we explore patterns across different populations and contexts (e.g., across samples that vary in developmental stage, gender, and country), as the extent to which a factor is “risky” for an outcome can vary substantially, such as by sex, gender, and developmental stage [10]. Only a few studies have investigated risk factors for emotional symptoms in early adolescence, and there is sparse evidence for girls specifically, despite their vulnerability. Furthermore, the extent and quality of evidence varies across different theorised risk factors. For example, studies examining associations between special educational needs (SEN) and symptoms are scarce and typically focus on specific conditions and small samples. Past investigations have often focused on single risk factors, failing to control for the confounding effects of other factors, despite evidence that risk factors co-occur. [10, 14, 15]. In this secondary data analysis, we addressed these major gaps by examining the effects of multiple risk variables. Specifically, we assessed eight candidate risk factors within our dataset for which there were varying levels of theoretical and/or empirical precedent of a relationship with higher levels of emotional symptoms:

- *Young relative age*: There is some evidence of greater symptoms among those youngest relative to peers in their academic year [14, 15], likely due to differences in schooling experiences [17]. However, evidence is limited with only one study focusing specifically on UK-based early adolescents [16].
- *Low academic attainment*: Research indicates an association between low academic attainment and symptoms, thought to be the result of self-perceived failure [18, 19]. Evidence suggests this relationship is stronger among girls [18, 20], perhaps due to growing discourses of girls as naturally academic, necessitating replicative work to build a robust cumulative evidence base.
- *High academic attainment*: Conversely, feminist theory notes that *high* achievement and/or cognitive ability could be problematic for girls and women due to increased pressure [21], but empirical investigation has been scant [22].
- *SEN*: SEN (e.g., moderate learning difficulties, speech, language and communication needs, and Autistic Spectrum Disorder) status has been shown to be related to emotional symptoms, partially due to stress caused by challenges in navigating education and peer relationships [23, 24]. However, evidence is based primarily on small samples with specific SEN conditions, limiting generalisability. There is some evidence of greater effects for girls than for boys in samples with specific conditions, such as dyslexia [24], but evidence about SEN as a broad category is lacking.
- *Low family income*: Evidence consistently indicates a relationship between low income and symptomatology, with multiple possible mediating pathways including poverty-related stress [11]. A range of effect sizes have been reported, depending on specific population characteristics including sex and gender [25], warranting further investigation.
- *Caregiving responsibilities*: A small number of research studies have suggested that young people providing emotional and physical caregiving typically performed by an adult may be at greater risk of mental health difficulties, potentially due to unmet needs or associated stress [26]. Investigation has, however, been hindered by the small proportion of those with caregiving responsibilities within the general population and difficulties in identifying such individuals, while findings are often specific to caregiving around specific conditions/circumstances.
- *Adversity*: Much of the research exploring adversity focuses on ‘adverse childhood experiences’ (ACEs), characterised by family dysfunction and childhood maltreatment. Evidence shows associations between such experiences in childhood and adolescence and adult symptoms, understood to be due to chronic stress. Research in adolescence is limited [27].
- *Neighbourhood socioeconomic deprivation*: Neighbourhood socioeconomic deprivation, comprising dimensions including low household income, low levels of education, and overcrowding, correlates with emotional symptoms in childhood and adolescence, potentially due to increased stressors including lack of resources, inadequate housing, and violence [28]. Those in deprived neighbourhoods are often exposed to a greater number of other risk factors, which can produce compound effects and necessitates ongoing examination [29].

Beyond identifying specific risk factors, it is also important to explore how cumulative risk exposure (CRE) relates to outcomes. Cumulative risk theory [14, 15] posits that the more risk factors one is exposed to, the greater the negative effects on outcomes, and that the *number* of risk factors, rather than their *nature,* best predicts outcomes. Researchers have theorised that the impact of CRE could be attributable to chronic stress, mediational mechanisms (e.g., maternal responsiveness) and/or disruption of proximal development systems [10]. Methodologically, this is assessed by identifying sample-specific risk factors, dichotomising (1 = risk present, 0 = risk absent) and summing these additively to create an unweighted composite score of the number of risk factors to which each individual is exposed [10]. Studies show associations between CRE and worsened outcomes, including some evidence for concurrent and longitudinal emotional symptoms and internalising difficulties [18, 30]. However, like risk factors, evidence suggests CRE is contextually specific, with evidence indicating that effects vary across populations according to characteristics such as sex, gender and age [10]. For instance, evidence indicates that CRE in early childhood may be particularly meaningful for concurrent and longitudinal outcomes [10]. However, CRE studies examining emotional symptoms have rarely focused on early adolescence, and Evans et al. [31] have previously highlighted the importance of evidence relating to CRE at this developmental stage given the shifts and vulnerability it encompasses. Furthermore, associations have not been examined specifically among early adolescent girls, despite a need to do so.

### Aims

Given the above, we set out to: (a) investigate the risk factors associated with emotional symptoms among girls aged 11–12 years, examining these jointly to isolate their unique contributions; and (b) assess whether exposure to a greater number of risk factors corresponds to higher levels of symptoms in this population. Such investigation contributes to knowledge by isolating unique risk associations with emotional symptoms, overcoming various methodological challenges present in prior evidence, and offering population-specific estimates of risk within a vulnerable group. We focus on girls specifically, rather than seeking to establish sex or gender differences, given consistent evidence of high rates of symptoms among girls and women and indications of early adolescence as a vulnerable period. Thus, investigation of the particular factors contributing to symptoms among early adolescent girls offers insights into a specific phenomenon within a vulnerable group, rather than offering a “comparative” analysis.

## Methods

### Context of the study

We draw on baseline data collected in 2017 for the evaluation of HeadStart, a large-scale programme exploring ways to improve young people’s mental health and wellbeing. HeadStart is an integrated programme in which local authorities and services across disadvantaged areas of England adopt a range of different approaches and interventions focusing on facilitating emotional resilience, responding to early signs of common mental health problems, and providing additional joined-up support where needed. Use of secondary data offers several strengths; this dataset comprises a variety of explanatory variables and a large sample spread across a range of settings in England. We also note a key limitation inherent to all secondary analyses: as study variables were predetermined, we were unable to capture all factors of potential interest (e.g., biological factors, such as adrenal hormones).

### Participants

The sample comprised 8327 girls aged 11–12 years (*M* [Mean] = 12.04, *SD* [standard deviation] = 0.29) across 100 English education settings. All Year 7 pupils in participating settings were invited to take part in the quantitative evaluation of HeadStart (Year 7 is the seventh year of compulsory education in England, when pupils are aged 11–12 years). Opt-out parental consent was used, and 114 of the Year 7 girls’ parents/carers opted their child out of the survey (as a result demographic information for these individuals was not available to explore differences in respondents versus those who were opted out). The current study makes use of data from all girls in this year group who took part in self-report data collection for the HeadStart evaluation. Ethnicity was similar to the national secondary school composition [32]; most participants were White (*n* = 6217; 75.9%), followed by Asian (*n* = 885; 10.8%), Black (*n* = 472; 5.8%), mixed (*n* = 344; 4.2%), other (*n* = 131; 1.6%), and Chinese (*n* = 15; 0.2%). The remaining 1.5% (*n* = 122) had incomplete ethnicity information. Free school meal (FSM) eligibility (*n* = 1436; 17.2%), a statutory benefit for school-aged children in England if their parents are classified as having low income, was higher than national levels (14% [32]).

### Data collection measures and procedures

Participants reported on their own emotional symptoms and on whether or not they have caregiving responsibilities. They provided this information as part of a wider self-report data collection procedure for the evaluation of HeadStart, as part of a broader inventory of mental health and wellbeing measures. These surveys were administered online in a teacher-facilitated session in participating schools, at a point convenient to the school in the period of March–July 2017. Data for remaining risk variables were obtained from the National Pupil Database (NPD) and were recorded as being up to date as of Spring 2017. This included gathering sex data for participants, which was used to determine their inclusion in the current study. Our focus on gender as a concept in the study relies on an imperfect proxy by drawing on sex data; however, in the absence of more inclusive gender data we sought to be sensitive to the ethical ramifications of implying attributions to sex and biological difference [33].

#### Emotional symptoms

The self-report Strengths and Difficulties Questionnaire (SDQ) emotional symptoms subscale [34] was used, which captures feelings of sadness and worry. There are five items: “I get a lot of headaches, stomach-aches or sickness”; “I worry a lot”; “I am often unhappy, down-hearted or tearful”; “I am nervous in new situations; I easily lose confidence”; and “I have many fears, I am easily scared” [34] (p.126). Items have three response options: “not true” (0), “somewhat true” (1), and “certainly true” (2). Summed scores range from 0–10, with higher scores indicating greater symptoms. Research has indicated acceptable psychometric properties for this subscale [35]. Here, Cronbach’s α was 0.72 and confirmatory factor analysis indicated acceptable fit: χ^2^ (5) = 255.28, *p* < 0.001; root mean square error of approximation (RMSEA) = 0.08, 90% CI [0.07, 0.09], *p* < 0.001; comparative fit index (CFI) = 0.98, and Tucker–Lewis Index (TLI) = 0.95.

#### Risk variables

Table 1 shows the measure used for each candidate risk factor, along with the approach to dichotomising data; all risk variables were obtained from the NPD, except caregiving responsibilities, which was self-reported.

**Table 1:** Measurement of candidate risk factors

Candidate risk factor	Measurement	Timing
Young relative age	Three categories based on birth month: youngest (May–August; n = 2921; 35.1%), middle (January–April, n = 2565; 30.8%) and eldest (September–December; n = 2810; 33.7%), with youngest and middle groups assessed as risk variables Timing: obtained Spring 2017; age not subject to change over time	Birth month was obtained in Spring 2017 and is not subject to change over time
Low and high academic attainment	Average point scores from key stage two statutory assessment tests. As both high and low attainment were assessed as risk factors it was necessary to isolate risk groups in advance of analysis; those in the lowest quartile were considered low attainers (*n* = 1902; 22.8%) and those in the upper quartile as high attainers (*n* = 1917; 23%)	Attainment recorded based on tests completed one year before self-report, in May 2016
SEN	Having SEN with or without a statement of SEN or Education, Health and Care plan (*n* = 609; 8.3%); note that this is inclusive within England’s approach whereby individuals can be formally recognised as requiring SEN support without necessarily having a statement or plan.^a^ Participants identified as having SEN due to mental health needs were excluded from this risk group given overlap with the outcome variable	Data records SEN status as recorded within the NPD in Spring 2017, alongside the self-report completion window
Low family income	Current or past FSM eligibility (*n* = 2982; 35.8%)	Data records eligibility at any point since May 2011 up until the most recent NPD census in Spring 2017, alongside the self-report window
Caregiving responsibilities	Participants responded yes or no to: “Young carers are children and young people under 18 who provide regular or ongoing care to a family member who has an illness, disability, mental health condition or drug/alcohol dependency. Are you, or have you ever been, a young carer?”	Completed at time of participant self-report (March-July 2017) and refers to both current and previous status
Adversity	Measured by proxy using binary Child in Need (CIN) status (*n* = 446; 5.4%), which captures elements of family dysfunction and childhood maltreatment and offers current information rather than relying on participant recall. A child or young person is considered ‘in need’ if it is deemed that: (a) They are not likely to achieve or to maintain reasonable health and development without local authority services and support, (b) their health and development is likely to be substantially impaired without local authority services and support, or (c) they have a disability. This may be due to factors including abuse or neglect, child disability, and family in acute stress. As this differs from the typical checklist approach used in ACEs research, we conceptualise this risk variable more generally as ‘adversity’ rather than adopting the specific concept of ACEs	CIN status is regularly reviewed by a social worker and is recorded annually; our data reflects whether CIN status was in place as of 31^st^ March 2017, which is concurrent with the self-report window
Neighbourhood socioeconomic deprivation	Income Deprivation Affecting Children Index score, a continuous variable where greater values denote greater deprivation^b^	This statistic is updated several times a year and our data reflects scores recorded in Spring 2017, alongside the self-report window

*SEN* special educational needs, *FSM* free school meals, *ACEs* adverse childhood experiences, *CIN* child in need

^a^Department for Education (2018c). Special educational needs: Transfer of statements of SEN to education, health and care plans, end March 2018. Ad hoc notice

^b^Risk variables treated as continuous in risk factor analysis (here, neighbourhood socioeconomic deprivation) found to be statistically significant risk factors are typically dichotomised to allow integration into a CRE index by isolating the upper or lower quartile as appropriate[10]

### Ethical considerations

Ethical approval was granted for the HeadStart evaluation by University College London’s ethics committee (reference 8097/003). Information sheets were provided to parents/carers and opt-out parental consent was used (114 girls opted out). Participants were presented with age-appropriate information and gave informed assent prior to completing by ticking a box to proceed.

### Statistical analysis

Analysis was undertaken using structural equation modelling in Mplus 8.1, using a robust weighted least squares (WLSMV) estimator to model emotional symptoms as a latent variable with categorical indicators [36]. As data were gathered from participants across 100 settings (mean cluster = 83), clustering was controlled for using Type = Complex (intracluster correlation coefficients = 0.00–0.40). RMSEA values below 0.06 and/or with 90% confidence intervals below 1.0, and CFI and TLI values above 0.95, indicated acceptable model fit [37, 38]. First, a linear multiple regression model was specified with risk variables predicting emotional symptoms. Variables were confirmed as risk factors where coefficients were positive and statistically significant (*p* < 0.05).

Next, confirmed risk factors were collated to create a CRE index, in line with guidance that a CRE index should comprise only empirically confirmed sample-specific risk factors (rather than all theorised variables) given the contextual specificity of risk [10]. Factors are coded as “1 = risk present” and “0 = risk absent” and summed (wherein a score of 1 denotes exposure to one risk factor, a score of 2 exposure to two risk factors, etc.). This index was then modelled as a predictor of symptoms to examine whether greater CRE is associated with increased symptomatology [10]. Risk factors were then added in turn as covariates to confirm that any effects were not driven by any one factor [10]. In risk factor and cumulative risk analysis, we followed MacKinnon et al. [39] in interpreting standardised beta coefficients (β) by using Cohen’s guidance [40] of 0.14, 0.39, and 0.59 as indicate of small, moderate, and large effect sizes, respectively.

To increase the interpretability of findings, and the degree to which the model fits the data, we performed a posterior predictive checking. The distributions of 1000 random datasets were simulated for each risk level based on the fitted model and were compared to the distributions of the real data, in line with statistical guidance from Gelman et al. [41]. The simulation was performed using the R package *rstanarm* (version 2.21.1) [42] and the findings were plotted using *bayesplot* (version 1.8.0) [43]. Using the recommended thresholds for high (score of 6) and very high (score of 7) emotional symptomatology [44], we calculated the proportion of scores that fell above those thresholds for both the simulated and real data.

## Results

### Preliminary analysis

No normality violations were identified. Missingness was low (2.3–3.0% for survey items and 0–7.2% for demographic variables). Little’s [45] missing completely at random test was significant (*p* < 0.001) and item-level missingness was predicted by SEN status and low academic attainment^2^; as such, data was presumed missing at random. As this level of missingness is generally considered acceptable with large samples and data assumed to be missing at random [46], this was not considered problematic. However, sensitivity analysis using maximum likelihood with robust standard error estimates (MLR), which uses full information, allowed confirmation that results were not affected by the use of the WLSMV estimator. Table 2 presents descriptive statistics.

**Table 2:** Descriptive statistics and bivariate correlations

Variable	*N*	*%*	1	2	3	4	5	6	7	8	9
1. Emotional symptoms ^a^	–	–	–								
2. Young relative age (youngest)	2921	35.1	.01	–							
3. Young relative age (middle)	2565	30.8	-.02	-.49***	–						
4. Low academic attainment	1902	22.8	.07***	.06***	.01	–					
5. High academic attainment	1917	23.0	-.05***	-.08***	.01	-.32***	–				
6. SEN	609	8.3	.06***	.03**	.01	-.28***	-.13***	–			
7. Low family income	2982	35.8	.07***	.00	-.01	-.17***	-.17***	.12***	–		
8. Caregiving responsibilities	1399	16.8	.12***	.03*	-.02	.10***	-.10***	.07***	.15***	–	
9. Adversity	446	5.4	.02***	.03*	-.02	-.07***	-.07***	.05***	.21***	.10***	–
10. Neighbourhood socioeconomic deprivation ^b^	–	–	.03*	.01	-.01	-.15***	-.15***	.10***	.37***	.11***	.15***

*SEN* special educational needs

^*^p < .05; **p < .01; ***p < .001

^a^M = 4.28, SD = 2.52 (range = 0–10), ^b^M = .24, SD = .14 (range = .01-.81)

### Risk factors

The first model in which the hypothesised candidate risk factors were modelled as predictors of emotional symptoms was shown to have a good fit: χ^2^ (41) = 321.03, *p* < 0.001; RMSEA = 0.03, 90% CI [0.03, 0.03], *p* = 1.00; CFI = 0.97, TLI = 0.95; MLR sensitivity analysis yielded similar results. Table 3 shows regression coefficients. Results showed four confirmed risk factors that were positively associated with emotional symptoms and statistically significant: (a) Low academic attainment with a small effect size, b = 0.06,^3^ β = 0.11, *p* < 0.01; (b) SEN with a small effect size, *b* = 0.08, β = 0.15, *p* < 0.01; (c) low family income with a small effect size, *b* = 0.05, β = 0.10, *p* < 0.01; and (d) caregiving responsibilities with a moderate effect size, *b* = 0.17, β = 0.33, *p* < 0.001. Neighbourhood socioeconomic deprivation was also statistically significant (*p* = 0.04) but was rejected as this relationship was negative and thus contrary to theoretical expectations (*b* = − 0.0.11, β = − 0.03, *p* < 0.05; see below). Three remaining candidate risk factors were rejected as they were not statistically significant: (a) Young relative age within academic cohort (both young [*p* = 0.58] and middle [*p* = 0.06] groups); (b) high academic attainment (*p* = 0.41); and (c) adversity (*p* = 0.60).

**Table 3:** Regression beta coefficients and standard errors for hypothesised candidate risk factors as predictors of symptoms (n = 7326)

	Unstandardised		Standardised	
Candidate risk factor	b	SE	β	SE
Young relative age (youngest)^a^	− 0.01	0.02	-.02	.04
Young relative age (middle)^a^	− 0.04	0.02	-.07	.04
**Low academic attainment**	0.06**	**0.02**	.11**	**.04**
High academic attainment	− 0.01	0.02	-.03	.04
**SEN**	0.08**	**0.02**	.15**	**.05**
**Low family income**	0.05**	**0.02**	.10**	**.03**
**Caregiving responsibilities**	0.17***	**0.02**	.33***	**.04**
Adversity	0.01	0.03	.02	.06
Neighbourhood socioeconomic deprivation	− 0.11*	0.05	-.03*	.02

Confirmed risk factors are shown in bold type

*SEN* special educational needs, *ACEs* adverse childhood experiences

^a^Eldest young relative age within academic cohort group (born September–December) utilised as reference category for dummy variables

^*^p < .05; **p < .01; ***p < .001

Further analysis was undertaken given the unexpected direction of the relationship between neighbourhood socioeconomic deprivation and emotional symptoms. Specifically, we used a bivariate structural equation modelling regression where the wider inventory of candidate risk factors were not included in the analysis as covariates, to assess whether inclusion of co-occurring risk factors (e.g., low family income) had resulted in the unexpected direction of this relationship. These results showed a relationship that *was* in the expected direction (*b* = 0.07; β = 0.02); however, this relationship was not statistically significant and the effect size negligible. As such this further analysis appeared to confirm that neighbourhood socioeconomic deprivation was not significantly associated with worsened symptoms in the study sample, both with and without controlling for wider risk exposure.

### Cumulative risk

**Fig. 1:**
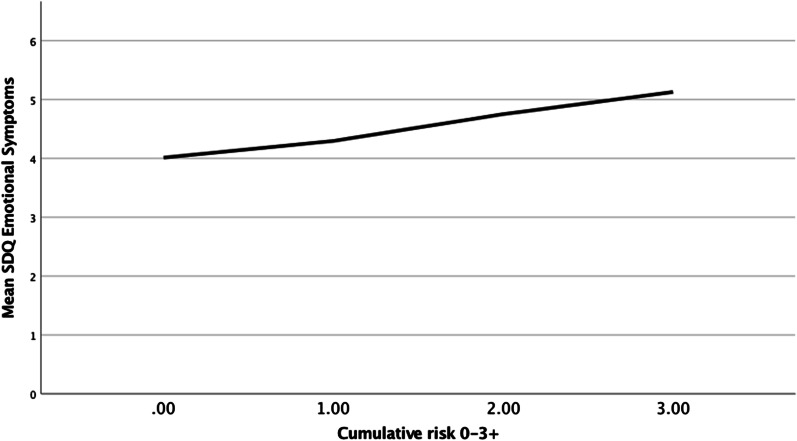
Line chart for emotional symptoms and the cumulative risk exposure (CRE) index

The four confirmed risk factors were summed to create a CRE index (*M* = 0.82, *SD* = 0.90). On an initial index ranging 0–4, less than one percent of participants (*n* = 69) had a score of 4, so the upper two categories were collapsed to create an index spanning 0–3 + , consistent with previous CRE research [10, 18]. The largest proportion of the sample presented no risk factors, with incrementally fewer participants represented at each level of exposure (45.3% = 0 risk factors; 33.0% = 1 risk factor; 15.7% = 2 risk factors; 5.9% = 3 + risk factors), meaning floor effects (45.3%) were present, consistent with previous studies [10]. No other normality violations were identified and missingness across the full index was low (0.2%). Figure 1 shows a line chart of the relationship between CRE and symptoms.

Next, the CRE index was modelled as a predictor of symptoms, with acceptable model fit: χ^2^ (9) = 430.25, *p* < 0.001; RMSEA = 0.08, 90% CI [0.07, 0.08], *p* < 0.001; CFI = 0.96; TLI = 0.93. MLR analysis yielded similar results. Results showed a statistically significant positive association between CRE and emotional symptoms (b = 0.09; β = 0.16; *p* < 0.001). In line with Cohen’s guidance [40], this standardised beta coefficient (β) indicates a small (but meaningful) relationship between CRE and self-reported symptoms. Inclusion of each covariate did not affect the significance of this relationship, suggesting that it was attributable to the CRE index [10].

### Simulated data

**Fig. 2:**
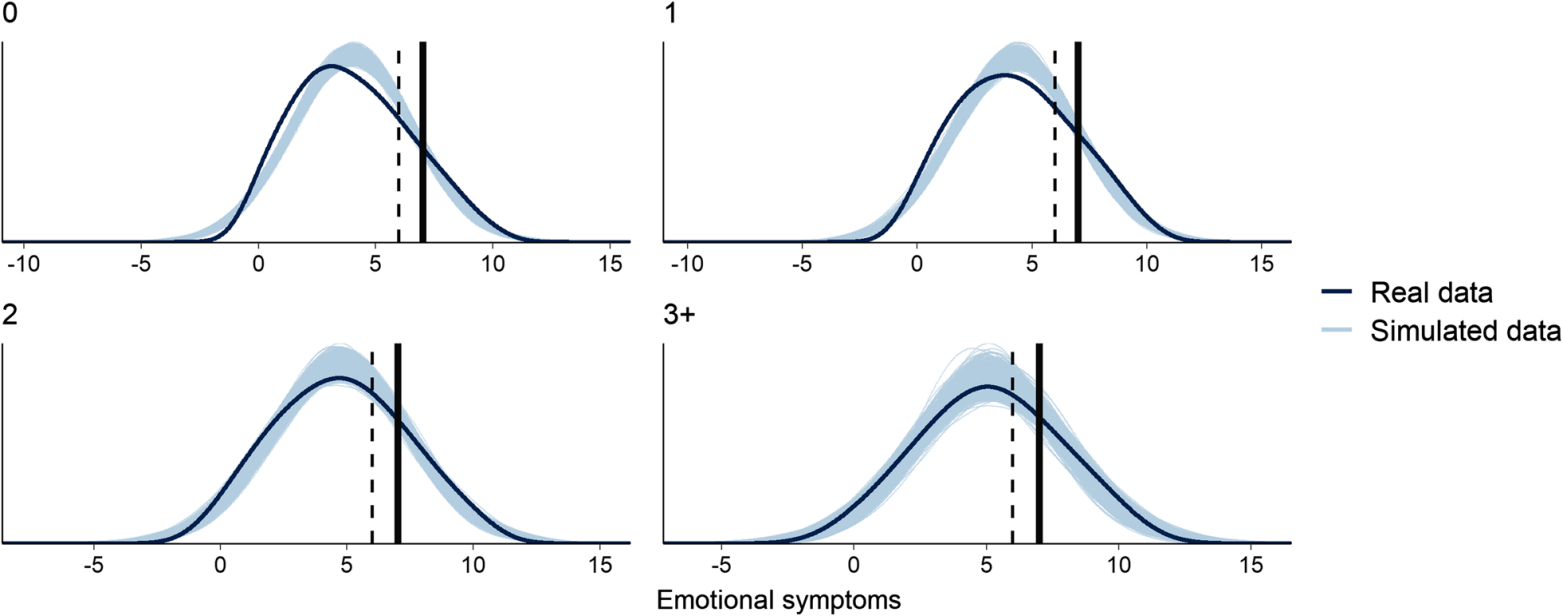
Distributions of simulated versus real data. *Note* Dashed line = high symptomatology (score ≥ 6); Straight line = very high symptomatology (score  ≥ 7)

The comparison of simulated distributions to those of the real data are shown in Table 4 and Fig. 2 for each level of cumulative risk. The distribution of real data was generally consistent with the simulated data, where an increase in CRE was associated with an increase in the proportion of individuals reporting high/very high emotional symptoms scores (in line with recommended thresholds [44]. For example, among the individuals identified as exposed to three or more risk factors, a much greater proportion report high/very high symptoms relative to those with no risks. A higher percentage of extreme scores was, however, observed in the real data.

**Table 4:** Proportions of high and very high emotional symptoms thresholds for real data and simulated data

		Real data		Simulated data	
Cumulative risk	*N*	High	Very high	High	Very high
0	3609	27.65	17.48	20.92	11.20
1	2565	30.80	21.21	25.05	14.26
2	1197	38.60	25.81	31.18	18.85
3 +	445	43.15	30.34	36.57	23.02

## Discussion

Our analyses identified four risk factors associated with emotional symptoms among early adolescent girls: low academic attainment, SEN, low family income, and caregiving responsibilities. In line with cumulative risk theory [10], greater levels of CRE corresponded to worsened symptoms. This research offers insight into the epidemiology of emotional symptoms among adolescent girls during a vulnerable period by offering evidence of the factors associated (and not associated) with symptoms among a large sample of girls. This offers a timely contribution to the wider knowledge and evidence given evidence of increased prevalence and a growing policy emphasis on understanding this phenomenon [47].

These findings build on previous research identifying risk factors in relation to emotional symptoms, offering evidence specific to early adolescent girls, and overcoming methodological issues that have limited prior evidence. Our inclusion of multiple factors allows isolation of the unique contributions (or lack thereof) each factor makes to symptoms among early adolescent girls. Furthermore, studies investigating heterogeneous circumstances, including SEN and caregiving responsibilities, have often focused on narrowed circumstances, thus offering highly contextual findings. Our examination of these factors as broader categories of experience offers more generalisable insight into potential effects of managing such circumstances more generally. For instance, evidence relating to associations between SEN and symptoms has often focused on specific conditions, such as dyslexia; given considerable heterogeneity both within SEN conditions and across SEN as a broad categorisation restricts the extent to which research into any one type of SEN can be generalised to others with the same or other conditions. Investigating the relationship between SEN as a broader category and emotional symptoms offers insight into the potential effects of navigating day-to-day life within an educational system and wider society that is often not congruent with one’s needs. Notably, prior investigation of caregiving responsibilities in relation to emotional symptoms has been rare; our finding that this variable was the strongest predictor warrants further research to explore population-specificity and offer qualitative insight.

Young relative age, high academic attainment, adversity, and neighbourhood socioeconomic deprivation were not significant as risk factors for emotional symptoms. As these variables were included based on theoretical and/or empirical precedence, findings perhaps offer further indications of the contextual nature of risk. For instance, the focus on *early* adolescence may be relevant; high academic attainment may be more problematic in later educational stages where high stakes examinations come into play. However, some aspects of our findings may also be methodological artefacts, which we explore here. Accounting for co-occurring risk variables may have offered more precise estimates than in previous research, while use of proxy measurement may have influenced results. For instance, we made use of Child in Need status to indicate adversity, which differs from the typical approach in the literature of focusing on ACEs conceptually and methodologically. ACEs research typically makes use of a checklist approach in which participants retrospectively indicate exposure to specific experiences, which may explain why our findings differ from wider evidence, as we did not use a standard ACEs measure, but instead relied on Child in Need status as a proxy for some experience of adversity. Future work should explore factors across varying circumstances and populations, including among girls across developmental stages.

Our findings offer evidence of small CRE effects in relation to early adolescent girls’ emotional symptoms. This was also supported by a posterior predictive checking and adds to growing evidence suggesting that CRE has negative implications for outcomes, including child and adolescent emotional symptoms [18, 30]. The small effect size observed in our sample is consistent with wider evidence relating to the association between CRE and emotional symptoms throughout childhood and adolescence, where small effect sizes are typically reported [30, 48–50]. However, these previous studies have typically focused on other developmental stages (e.g., middle childhood) or have spanned wide age ranges rather than focusing more narrowly on early adolescence. Thus, our study contributes evidence that the relationship between CRE and emotional symptoms appears small among girls in early adolescence specifically, as at other stages of childhood and adolescence, despite evidence of vulnerability among girls for emotional symptoms at that time. However, this is not to suggest that a small effect size is negligible in the context of emotional symptoms; at the population level, an increase or decrease in one or two points in a mental health survey can translate into meaningful differences to daily life [51, 52]. Our simulation analysis illustrates the shift occurring for those in the upper levels of CRE compared to those with little to no exposure. Future research should explore the meaning and impact of symptoms within adolescent girls’ day-to-day lives, and further examine associations between CRE and outcomes in early adolescence to explore longitudinal effects over time and to investigate whether there are differences in associations with CRE across genders and between outcomes.

Notably, the particular risk factors identified in the current study are each understood to affect mental health at least partially through the daily stress they introduce (e.g., see [53, 54]. This may reflect the theory that CRE leads to overwhelming stress levels, in turn impacting outcomes [10], and is consistent with the theory that chronic stress might explain gender differences in emotional difficulties [55]. These findings highlight a need to consider how support and treatment can be facilitated in a manner that is sensitive to the stressors affecting an individual and how such stressors operate in their daily lives, while also working to alleviate stressors where possible.

Our findings relate to factors associated with symptoms among a sample of girls reporting on their symptoms in 2017 (i.e., at a time where we know this population is experiencing increased symptoms), but do not *specifically* capture factors that may be directly associated with the increased symptomatology observed among adolescent girls in recent years. Potential explanations for this apparent increase remain poorly understood and are generally speculative; researchers have posited a range of factors that may be contributing to such an increase, such as aspects of social media usage [6], increased sexualisation of adolescent girls [6, 56], increased academic pressure [4, 56], and a lack of prioritisation of emotional symptoms in schools [6]. A priority in future research is engaging with adolescent girls themselves to build on researchers’ speculative explanations and to understand their perspectives on these issues, and explore ways in which these complex factors can be investigated in relation to time trends.

### Generalizability

There are limitations regarding the generalizability of these findings. First, our simulation showed some high values in the emotional symptoms experienced by our sample that do not appear in the replications (i.e., the simulated data, shown in light blue in Fig. 2). While this would suggest some caution in the interpretation of the SDQ categorisations (high versus very high) in our sample, the increase of symptomatology by cumulative risk was consistent between the real and simulated data. Secondly, although several candidate risk factors were measured prior to self-report of symptoms given that they are drawn from a wider database (NPD) which is regularly updated (except caregiving responsibilities, which was self-reported), causality cannot be established given the inability to control for prior symptoms. Future studies should adopt longitudinal designs to establish directionality. Third, although use of routinely recorded NPD data means risk information is relatively reliable, this also represents proxy variables for more complex phenomena. For instance, ACEs are typically measured using a cumulative checklist where older adolescent and adult participants identify themselves whether they have been exposed to specific childhood and adolescent experiences [57]. This is distinct from our use of a present/absent dichotomy that relies on formal recognition of these kinds of complex family circumstances, as captured within CIN status. As such, use of CIN status as a proxy for ACEs in the current study overcomes issues around reliance on recall that are typically found in ACEs research [57], but also offers narrow information and likely overlooks many individuals experiencing adversity given that it focuses on those in the most extreme circumstances. Though the summed index in CRE research means binary information is not considered problematic, it may be useful to explore risk factors using varied measurement to create cumulative evidence around the unique contributions of individual risk factors alongside others and within a cumulative risk index.

Fourth, although self-report is an appropriate means of measuring adolescent mental health, it can be subject to biases including social desirability; future research may benefit from a multi-informant approach. Furthermore, the SDQ emotional symptoms subscale captures a narrow grouping and range of symptoms, and the use of secondary data analysis meant we were unable to use a more comprehensive measure of symptoms. However, evidence suggests the SDQ emotional symptoms subscale shows good known groups validity in distinguishing between healthy samples and those with psychiatric disorder [58]. Cumulative risk theory and methods also have limitations. Though the additive approach mirrors the way risk factors co-occur, this is perhaps reductionist [59]; treating risks as equal is inconsistent with differential risk factor effects, while statistically collapsing variables may reduce predictive power [10, 59]. Finally, confirmation of only four risk factors here precluded more nuanced CRE investigation that would require a more extensive index (e.g., the functional form of the CRE-symptom relationship).

## Conclusions

This research highlights several factors within home and school life associated with emotional symptoms among early adolescent girls, namely academic attainment, special educational needs, low family income, and caregiving responsibilities. Moreover, findings show that where individuals are exposed to several such factors, symptoms are likely to be worsened. Findings demonstrate the need to identify girls experiencing stressful daily challenges and provide intervention and support, particularly given the apparently growing vulnerability to emotional symptoms among adolescent girls. In particular, targeted interventions may be valuable for those demonstrating emergent symptoms, as one component of a wider whole-school approach to mental health promotion [60]; for instance, school-based approaches frequently offered include individual or group counselling, interventions that aim to target specific symptoms such as low mood, and other interventions such as peer support strategies (e.g., see [61]). However, such actions should be sensitive to individual circumstance, because although varying risk profiles can similarly contribute to worsened outcomes, daily experiences may differ greatly. Future research should examine whether particular constellations of combined risk more greatly influence symptoms, and should also investigate underlying mechanisms for CRE effects.

## Data Availability

The HeadStart survey data on mental health and wellbeing belongs to the Evidence Based Practice Unit (a collaboration between UCL and the Anna Freud National Centre for Children and Families, AFNCCF), who led the HeadStart evaluation. The authors accessed this survey data via membership in a consortium involved with the HeadStart evaluation. As collaborators on the main HeadStart evaluation, the authors were granted secure remote access to this data by the principal investigator of the main HeadStart evaluation, Dr. Jessica Deighton. HeadStart data cannot be made publicly available, since consent was not obtained from participants for the public sharing of their survey responses. However, an anonymised version of the survey dataset used in the present paper is available on request from Dr. Jessica Deighton (Jessica.DeightonPhD@annafreud.org) or Dr. Tanya Lereya (Tanya.lereya@annafreud.org) under the following terms: 1. Schedule and arrange for site visit to AFNCCF to analyse data (password to user account supplied). 2. Analysis to be worked on in situ. 3. Results (but not data) taken away. In the event that either of these individual leaves the AFNCCF, updated contact information for new guardians of the data will be provided to BMC Women’s Health.

## Abbreviations

ACEs: Adverse childhood experiences
CFI: Comparative fit index
CRE: Cumulative risk exposure
FSM: Free school meals
*M*: Mean
MLR: Maximum likelihood with robust standard error estimates
NPD: National Pupil Database
RMSEA: Root mean square error of approximation
*SD*: Standard deviation
SDQ: Strengths and Difficulties Questionnaire
SEN: Special educational needs
TLI: Tucker-Lewis Index
WLSMV: Robust weighted least squares

## Footnotes

1 It should be noted that we focus conceptually on gender in the current study, rather than sex. Differences in emotional symptoms are understood to relate to both biological and psychosocial factors [55, 62], meaning that the specific approach appropriate for a given study is not straightforward in this area. However, in drawing on the literature we aim to make use of the language used in specific studies and reviews when discussing their conclusions.

2 Demographic variables tested as possible predictors of missingness were: ethnicity, English as an additional language, SEN status, low academic attainment, low family income, experience of adversity, young relative age, having caregiving responsibilities, and neighbourhood socioeconomic deprivation.

3 *b* denotes the unstandardised beta coefficient, and β denotes the standardised beta coefficient.
